# A Successful Pregnancy Outcome Using Laser-Assisted Hatching and Platelet-Rich Plasma Perfusion in Advanced Maternal and Paternal Age: A Case Report

**DOI:** 10.7759/cureus.63926

**Published:** 2024-07-05

**Authors:** Jafar S Shaikh, Akash More, Nancy Nair, Jarul Shrivastava, Charu Pareek

**Affiliations:** 1 Clinical Embryology, Datta Meghe Medical College, Datta Meghe Institute of Higher Education and Research, Nagpur, IND; 2 Clinical Embryology, Datta Meghe Institute of Higher Education and Research, Wardha, IND

**Keywords:** intracytoplasmic sperm injection, laser-assisted hatching, platelet-rich plasma-perfusion, in vitro fertilization, assisted reproductive technology

## Abstract

Infertility affects millions globally, with advanced parental age posing a significant risk. This case report details a couple who experienced secondary infertility for 12 years. Following multiple unsuccessful attempts at assisted reproductive technology (ART), a personalized treatment regimen involving platelet-rich plasma (PRP) perfusion and laser-assisted hatching (LAH) resulted in a successful pregnancy. Diagnostic evaluations identified specific reproductive challenges, leading to tailored interventions. A positive pregnancy outcome was achieved after PRP treatment enhanced endometrial thickness and LAH facilitated embryo implantation. This case highlights the importance of individualized treatment strategies in infertility management and proves the potential efficacy of PRP and LAH in overcoming recurrent implantation failure. Further research is needed to explain the roles of PRP and LAH in improving pregnancy outcomes, especially in older parents and couples with a history of failed in vitro fertilization (IVF) treatments.

## Introduction

Approximately 17.5% of adults, or about one in six people globally, face infertility, highlighting the critical need to expand access to affordable, high-quality fertility care for those affected. Infertility is defined as the failure to conceive following a year of continuous unprotected sexual intercourse [1]. The most common type of infertility among women worldwide is secondary infertility, which is frequently brought on by infections of the reproductive system. It is commonly observed in regions with inadequate maternity care and abortion practices, which are often linked to a higher risk of infection [2].

The age of conception is rising as a result of modern living. Advanced maternal age (AMA >35 years) is a significant risk factor for infertility in women. Women in their advanced maternal age are more likely to experience unfavorable obstetrical and perinatal outcomes [3]. As crucial as the oocyte is for normal fertilization, a successful pregnancy outcome is significantly influenced by the factors that maintain the endometrium and support implantation. Overall, endometrial function, including female fertility, is affected by AMA [4].

It is also known that sperm quality and quantity decline with advancing paternal age leading to higher incidence of miscarriages [5]. A majority of evidence reports an age-related decline in semen volume, motility, and proportion of morphologically normal sperm and advanced paternal age effects on sperm DNA fragmentation [6].

A functioning embryo, a receptive endometrium, and a coordinated interaction between the blastocyst and endometrium are needed for successful implantation. Factors that influence and provide successful pregnancy outcomes are platelets. Platelets from peripheral blood are concentrated into platelet-rich plasma (PRP), an autologous blood product that contains a significant amount of cytokines and growth factors [7]. Research has demonstrated that PRP is rich in growth factors that enhance angiogenesis, cell proliferation, and differentiation. By promoting angiogenesis, PRP increases blood flow to the endometrium, thereby improving its thickness, vascularity, and receptivity, aiding in embryo implantation [8]. PRP has been extensively employed across multiple medical specialties as a type of regenerative medicine [9]. Research studies have shown that autologous PRP can expand and repair the thin endometrium, reducing the risk of implantation failure and improving the quality of pregnancy outcomes [10]. The benefits of autologous PRP therapy for endometrium treatment are to enhance endometrial thickness and pregnancy outcomes [11,12]. PRP contains various proteins, numerous growth factors, and cytokines within platelets, which are administered intrauterine. These components impact the endometrium by encouraging cell proliferation and exhibiting anti-inflammatory properties, thereby facilitating successful implantation [11].

As a solution, laser-assisted hatching (LAH) is a commonly used method in assisted hatching due to its benefits, such as brief exposure time, straightforward procedure, precise positioning, indirect contact, safety, and efficiency [12-14]. In LAH, an infrared diode laser is used to generate a hole in the embryo’s zona pellucida (ZP), increasing the embryo's chances of successfully implanting in the receptive endometrium. The procedure is ideally carried out on day three of embryo development [15].

## Case presentation

Patient information

The couple, experiencing infertility for 12 years, visited an assisted reproductive technology (ART) clinic located in Maharashtra. The male patient was 39 years old, and the female was 36 years old. The couple had no history of addictions to alcohol, tobacco, or smoking. Both of them received a detailed explanation of the processes, benefits, and drawbacks, and their informed permission was acquired.

Medical history

The female patient had previously experienced one miscarriage after two years of marriage after having sexual intercourse. The prior incidence of fertilization and implantation indicated that the couple has a case of secondary infertility. With an unsuccessful pregnancy, the couple underwent three unsuccessful intrauterine insemination (IUI) attempts, two failed cycles of in vitro fertilization (IVF), and two failed attempts at frozen embryo transfer (FET). This medical history highlights the complexity of their infertility struggles and the need for specialized treatments to enhance their chances of conceiving.

Clinical findings and investigations

Extensive diagnostic evaluations, including hormonal assessments, ovarian reserve testing, and measuring the endometrial thickness, were conducted for the husband and wife accordingly. The results indicated a personalized treatment plan to address their specific fertility challenges. The hormonal profile of the female patient is shown in Table 1.

**Table 1:** Hormonal profile of female AMH: anti-müllerian hormone; FSH: follicle-stimulating hormone; LH: luteinizing hormone; IU: international units; ng: nanogram; pg: picogram; mL: milliliter

Parameters	Findings	Reference value
LH	10.1	5 IU/L
FSH	5.1	10 IU/L
AMH	0.9	0.8-1.0 ng/mL
Estradiol	141	30 to 400 pg/mL

Timeline

A couple who had been married for 12 years experienced secondary infertility. They had previously experienced one miscarriage after having sexual intercourse, two years after their marriage. Their initial attempt at IUI took in October 2021, resulting in an unsuccessful outcome. Subsequently, the other two IUI in December 2021 and February 2022 were pursued unsuccessfully, after which the patient went through two IVF cycles in November 2023, and both cycles failed. With two unsuccessful IVF cycles, the couple next underwent two FET procedures in Jan 2024, both of which reported negative. This prompted further hormonal and PRP intervention.

Therapeutic intervention

**Figure 1:**
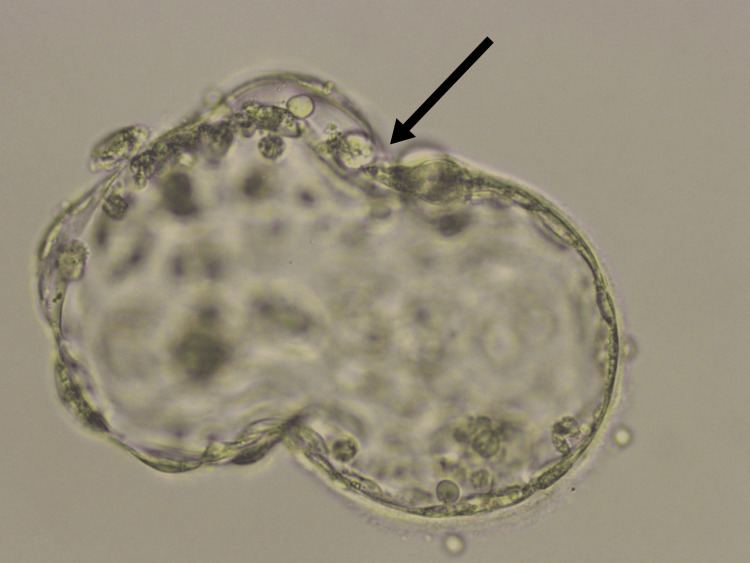
Blastocyst hatched by LAH Black arrow shows hatched blastocyst, LAH - laser assisted hatching

Gonadotropin-releasing hormone (GnRH) antagonist protocol was opted to regulate the timing of ovulation and promote the development of multiple follicles in the ovaries. After 14 days of ovarian stimulation, we proceeded with the ovum pick-up (OPU). A GnRH agonist trigger was administered, and ovarian aspiration was scheduled 36 hours post-trigger. During the OPU, we retrieved five high-quality metaphase II oocytes and four metaphase I oocytes. On the same day, intracytoplasmic sperm injection (ICSI) was performed, resulting in the formation of four high-quality cleavage-stage embryos. PRP treatment was administered on day eight before ET to enhance the endometrium. Following the PRP treatment, a significant increase in endometrial thickness was observed, from 6.5 mm to 8.7 mm, on day 10 of the menstrual cycle. Before ET, a laser-assisted hatching procedure was performed to improve implantation outcomes. The decision to proceed with FET was made after thorough consultation with the female partner and obtaining detailed informed consent. On the day of ET, two high-grade laser-hatched embryos were transferred. PRP treatments and laser-assisted hatching were integral to this treatment plan, aimed at enhancing endometrial receptivity and improving implantation success. Figure 1 shows a blastocyst hatched by LAH.

Follow-up and outcome

Throughout the procedure, the female experienced no difficulties. The patient was instructed to avoid heavy lifting and to perform moderate exercises. The human chorionic gonadotropin (β-hCG) level was tested 14 days after FET, and this time, the result revealed a positive sign for pregnancy, with a level of 1131 mIU/mL. The female was then closely observed for the duration of her pregnancy to make sure she followed instructions for taking the prescribed medication.

## Discussion

The present study demonstrates an example of the successful use of PRP perfusion and laser-assisted hatching as an effective method for overcoming a couple's difficulties associated with recurrent implantation failure. This illustrates the significance of selecting appropriate technology according to the individual demands of the patient. Understanding the factors that contribute to the failure of implantation and modifying treatments accordingly can greatly improve the chances of a successful pregnancy for infertile couples.

PRP has the potential to be promising in reproductive medicine through the demonstration of endometrial regeneration, restoration of the menstrual cycle, improving folliculogenesis, increasing endometrial receptivity, and increasing clinical pregnancy and live birth rate [16]. The therapeutic benefits of autologous PRP for endometrial development in women with thin endometrial tissue. In the trial by Chang et al., five women received diagnosis with unsustainable endometrium and had not responded adequately to standard therapy throughout the cycle received PRP infusions. All five women reported satisfactory reactions to treatment, and four of them reported normal pregnancies [13].

In recent years, a lot of research has focused on autologous PRP after infertility. PRP has been shown to affect tissue regeneration, angiogenesis, cell migration, differentiation, and proliferation. These functions are aided by the various cytokines and growth factors that PRP creates upon activation [14,16]. Transforming growth factor-beta, fibroblast growth factor that resembles vascular endothelial cell growth factor, and epidermal growth factor are a few examples of growth factors and cytokines [14,16]. Aged patients, those with frozen-thawed embryos, and those who have experienced recurrent in vitro fertilization-embryo transfer failures have all had the option of assisted hatching [17]. In assisted hatching, the zona pellucida is purposefully disturbed, which was first proposed in 1996 [18]. In a similar way, Kutlu and colleagues study revealed that like enhancing the ET process, endometrial receptivity, and the embryo's capacity for implanting have been proposed and implemented in practice in an effort to raise the implantation rate [19].

The Hammadeh et al. trial provided evidence that assisted hatching (AH) was beneficial in specific situations, particularly for women over 38 and for certain inpatient groups, despite generally unfavorable results [15]. After AH, there was a rise in the incidence of clinical pregnancy and implantation. The laser hatching used in the case study was based on research conducted by Hammadeh et al. The limitation of this report is that it is a single case, making it impossible to generalize the findings to the entire population. It's also critical to remember that ARTs are always changing, and methods like laser hatching are the subject of continuous study and improvement.

This case study provides one example of evidence that using PRP in conjunction with tempol medicine may be a ground-breaking method for improving thin endometrium and raising the chances of succeeding in clinical pregnancy through ART.

## Conclusions

The effective application of LAH on the embryo prior to ET is described for a patient who had secondary infertility, a history of miscarriage, and multiple implantation failures. The case further highlights how PRP and LAH may benefit individuals who have experienced repeated implantation failure. The results imply that certain ART difficulties may be addressed by procedures like PRP and LAH and the importance of individualized approaches in evolving reproductive technologies.
